# Supplementary material for: Bioinformatics Identification of TUBB as Potential Prognostic Biomarker for Worse Prognosis in ERα-Positive and Better Prognosis in ERα-Negative Breast Cancer
Source: Diagnostics (Basel). 2022 Aug 26;12(9):2067. doi: 10.3390/diagnostics12092067 (PMC9498198; doi:10.3390/diagnostics12092067)
Supplement: Supplementary file 1 [file diagnostics-12-02067-s001.zip › Supplementary Tables S2 and S3.pdf]

Supplementary Table S2. Shows the genes that correlate positively and negatively with *TUBB* in ERα-positive breast cancer patients

Gene symbol	Pearson's correlation coefficient	p-value	No. patients
TUBBP1	0.7746	< 0.0001	530
LSM2	0.6182	< 0.0001	3685
STMN1	0.6153	< 0.0001	3685
NRM	0.6045	< 0.0001	3685
TUBA1C	0.5972	< 0.0001	3685
KIFC1	0.5935	< 0.0001	3685
KIF2C	0.5928	< 0.0001	3685
TCF19	0.5919	< 0.0001	3685
MYBL2	0.5869	< 0.0001	3685
ORC1	0.5816	< 0.0001	3685
ABCF1	0.5815	< 0.0001	3685
TUBA1B	0.58	< 0.0001	3685
CDC20	0.579	< 0.0001	3685
RAD54L	0.5679	< 0.0001	3685
CDCA8	0.5637	< 0.0001	3685
MCM10	0.5631	< 0.0001	3685
TUBA5P	0.496	< 0.0001	530
CFL1	0.491	< 0.0001	3685
TMSB10	0.472	< 0.0001	3685
NUDT1	0.4657	< 0.0001	3685
SHCBP1	0.4589	< 0.0001	3685
GINS3	0.4468	< 0.0001	3685
CDC6	0.4441	< 0.0001	3685
CLIC1	0.4399	< 0.0001	3685
SAPCD2	0.4368	< 0.0001	3685
SAE1	0.4364	< 0.0001	3685
SUV39H1	0.4249	< 0.0001	3685
CENPM	0.4228	< 0.0001	3685
PSMD2	0.4178	< 0.0001	3685
DTYMK	0.4148	< 0.0001	3685
NRBP1	0.4121	< 0.0001	3685
CENPU	0.4102	< 0.0001	3685
COLGALT1	0.4066	< 0.0001	3685
PPIAP22	0.406	< 0.0001	530
EME1	0.4059	< 0.0001	3685
ECE2	0.4052	< 0.0001	3685
ACTG1	0.4047	< 0.0001	3685
TUBAP2	0.4044	< 0.0001	530
HNRNPAB	0.4036	< 0.0001	3685
ATP5MC1P4	0.4015	< 0.0001	530
ACTB	0.4011	< 0.0001	3685
PRC1-AS1	0.4008	< 0.0001	3362
AIDAP2	-0.4325	< 0.0001	530
CBX7	-0.4316	< 0.0001	3685

CREBRF	-0.4274	< 0.0001	3685
APH1B	-0.4222	< 0.0001	3685
CYB5D2	-0.4135	< 0.0001	3685
FRY	-0.412	< 0.0001	3685
SGMS1-AS1	-0.4099	< 0.0001	530

Supplementary Table S3. Shows the genes that correlate positively and negatively with *TUBB* in ERα-negative breast cancer patients

Gene symbol	Pearson's correlation coefficient	p-value	No. patients
CHEK1	0.6477	< 0.0001	510
MDC1	0.6334	< 0.0001	510
BAG6	0.6324	< 0.0001	510
GMNN	0.6234	< 0.0001	510
DEK	0.6234	< 0.0001	510
HDAC2	0.6091	< 0.0001	510
CCHCR1	0.6033	< 0.0001	510
SRPK1	0.6028	< 0.0001	510
FANCE	0.5892	< 0.0001	510
DHX16	0.5834	< 0.0001	510
EHMT2	0.5791	< 0.0001	510
DAXX	0.5783	< 0.0001	510
PSRC1	0.5778	< 0.0001	510
USP1	0.5751	< 0.0001	510
ATAT1	0.5748	< 0.0001	510
MSH2	0.5704	< 0.0001	510
NUF2	0.5693	< 0.0001	510
PRR3	0.5622	< 0.0001	510
SKP2	0.5596	< 0.0001	510
RNF8	0.5553	< 0.0001	510
DNA2	0.5527	< 0.0001	510
MMS22L	0.5518	< 0.0001	510
ILF2	0.5487	< 0.0001	510
SNRPA	0.5476	< 0.0001	510
RIOK1	0.5475	< 0.0001	510
MSH6	0.5408	< 0.0001	510
E2F7	0.534	< 0.0001	510
CENPL	0.5002	< 0.0001	510
PBK	0.4955	< 0.0001	510
ERI3	0.4931	< 0.0001	510
DDIAS	0.4771	< 0.0001	510
KDM2B	0.4735	< 0.0001	510
C1orf112	0.441	< 0.0001	510
CHCHD3	0.4275	< 0.0001	510
BCL11A	0.4164	< 0.0001	510
PIP	-0.5188	< 0.0001	510
FMO5	-0.5053	< 0.0001	510

TMBIM4	-0.4999	< 0.0001	510
XBP1	-0.4996	< 0.0001	323
SCUBE2	-0.4937	< 0.0001	510
EFCC1	-0.4853	< 0.0001	510
ABCC11	-0.4823	< 0.0001	510
FAM214A	-0.4823	< 0.0001	510
SLC7A8	-0.4787	< 0.0001	510
LOC100505942	-0.432	< 0.0001	241
RETSAT	-0.4088	< 0.0001	510
PLAAT2	-0.4085	< 0.0001	510
LINC00160	-0.4076	< 0.0001	428
LIN7A	-0.4068	< 0.0001	510
ADH1C	-0.4006	< 0.0001	510
